# Atypical central neurocytoma with anaplastic histological features in the lateral ventricle of a 37-year-old man

**DOI:** 10.17879/freeneuropathology-2026-9697

**Published:** 2026-08-17

**Authors:** Mari Valkonen, Paula Bendel, Tuomas Rauramaa, Susanna Rantala

**Affiliations:** 1 Department of Clinical Pathology, Kuopio University Hospital, Kuopio, Finland; 2 Unit of Clinical Pathology and Forensic Medicine, Institute of Clinical Medicine, University of Eastern Finland, Kuopio, Finland; 3 Department of Clinical Radiology, Kuopio University Hospital, Kuopio, Finland; 4 Neurosurgery KUH NeuroCenter, Institute of Clinical Medicine, Kuopio University Hospital, University of Eastern Finland, Kuopio, Finland

**Keywords:** Atypical central neurocytoma, Lateral ventricle tumor, DNA methylation profiling, Copy number alterations

## Abstract

We report a case of atypical central neurocytoma with anaplastic histological features in the lateral ventricle of a previously healthy 37-year-old man. Histologically, the tumor exhibited anaplastic cell morphology, necrosis, increased mitotic activity, and vascular proliferation. The tumor had copy number alterations, but methylation profiling did not provide a match to any known methylation class, and mutation analyses were negative for pathogenic alterations. Atypical central neurocytoma is a challenging and rare diagnosis, and the World Health Organization (WHO) Classification of Tumors has not yet clarified diagnostic criteria for this putative entity. In a literature review, however, atypical central neurocytoma showed increased aggressiveness compared to conventional central neurocytoma, WHO grade 2.

## Introduction

Central neurocytoma is an uncommon brain tumor that generally arises within the lateral ventricles or the third ventricle near the foramen of Monro. Unlike many other central nervous system tumors, central neurocytomas lack defining molecular features. Neurocytomas generally have a favorable prognosis, although recurrences can occur. Single cases of craniospinal dissemination have been reported (1,2). General histopathological features include monotonous, uniformly round cytomorphology with a neuronal immunophenotype and a low proliferation index. While necrosis, histological atypia, and vascular proliferation have been reported rarely in central neurocytomas, these features have not been significantly associated with prognosis (3). More aggressive behavior has been reported in neurocytomas with a higher Ki-67 proliferation index (> 2–3 %) and increased mitotic counts (3). The current WHO Classification of Tumors categorizes central neurocytomas as grade 2, although it acknowledges that some tumors may harbor a higher proliferation index and higher-grade histological features, suggesting that such neurocytomas should be referred to as atypical central neurocytomas. Methylation classification currently cannot differentiate conventional and atypical central neurocytomas, although the data on methylation profiling of atypical central neurocytomas are still quite limited (6). Furthermore, the cutoffs for proliferation between these tumors are under debate.

In general, there is scarce evidence on consistent genetic alterations in central neurocytomas, and their mechanism of pathogenesis remains unknown. Loss or abnormal immunohistochemical staining for H3K27me3 has been reported in neurocytomas, suggesting that epigenetic alterations in H3K27me3 may participate in tumorigenesis in central neurocytomas (7). Central neurocytomas have been reported to harbor multiple copy-number aberrations (8), and methylation profiling has aided in diagnosis and differentiation between histologically similar tumors (9).

In this report, we discuss a case of an unusual intraventricular tumor in a 37-year-old man. Neuroradiological findings indicated a central neurocytoma, but the histological findings were unusually anaplastic, and methylation classification did not categorize this tumor. Immunohistochemical staining for H3K27me3 indicated partial loss. Methylation profiling revealed an excess number of copies on multiple chromosomes. Genetic analysis did not provide additional information on this tumor.

## Case report

**Figure 1:**
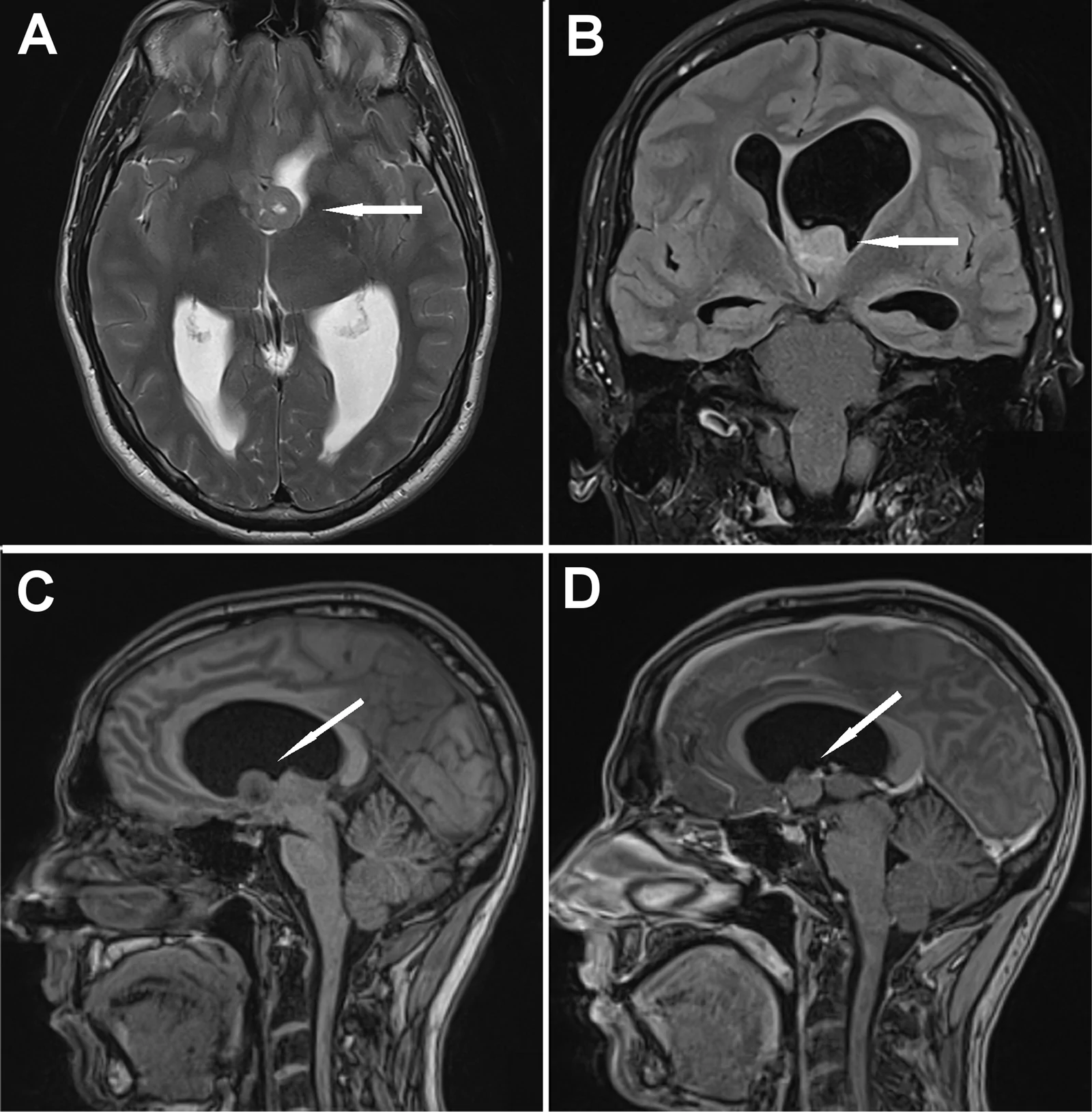
**Figure 1**. Neuroradiological features of this case of central neurocytoma. **A:** T2-weighted images, axial view. Moderately well-defined solid tumor (arrow) with small microcysts appears isointense relative to the cerebral cortex. **B:** FLAIR images in the coronal view. The tumor (arrow) shows hyperintensity compared to the brain. **C:** Non-contrast T1-weighted images in the sagittal view. The tumor (arrow) appears isointense compared to the gray matter. **D:** Contrast-enhanced T1-weighted images in the sagittal view. The tumor (arrow) shows mild-to-moderate, slightly heterogeneous enhancement.

A 37-year-old, previously healthy male sought treatment at the emergency unit. He had suffered from progressive headaches for two months and vomiting for the last few days. Brain computed tomography (CT) revealed a tumor in the left lateral ventricle, obstructive hydrocephalus, and midline shift to the right. The patient was transferred to the neurosurgery unit of our hospital. Brain CT and magnetic resonance imaging (MRI) revealed obstructive hydrocephalus due to a tumor measuring 18 × 15 × 16 mm, which was blocking the foramina of Monro at the base of the left frontal horn **(Fig. 1)**. The moderately well-defined solid tumor appeared isointense on T1/T2-images and fluid-attenuated inversion recovery (FLAIR)-hyperintense relative to the cerebral cortex. The tumor showed slight microcystic changes, with no calcifications or hemorrhages. Diffusion-weighted imaging showed mild restriction with apparent diffusion coefficient values of approximately 700. Tumor enhancement was mild and heterogeneous in nature. Considering the location and signal characteristics of the tumor, the neuroradiological working diagnosis was central neurocytoma.

The patient underwent surgery on the same day, and the tumor was resected using a left-sided frontal transcortical approach. The patient’s recovery was swift after the operation, and postoperative MRI showed no residual tumor.

**Figure 2:**
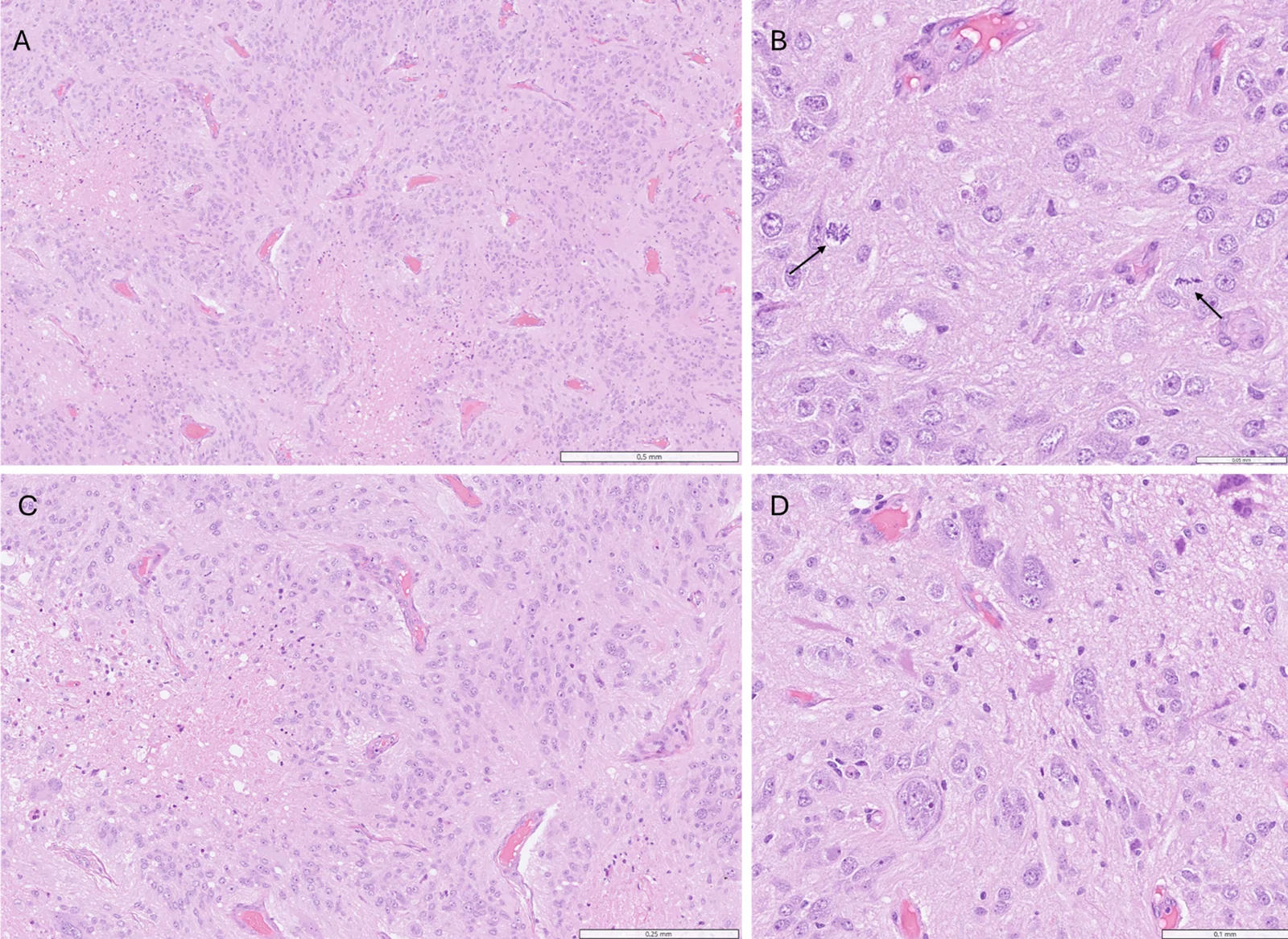
**Figure 2**. **A:** The tumor consisted of large tumor cells, geographic necrosis, and prominent vascularity (hematoxylin-eosin [H&E], 5x). **B:** Mitotic figures (arrow) were easily identified (H&E, 40x). **C:** Fibrillary matrix and branching vascularity were observed (H&E, 10x). **D:** Some tumor cells had anaplastic features, and occasional multinucleated cells were observed. A fibrillary matrix was occasionally observed (H&E, 20x).

The pathology unit received 15 × 15 × 7 mm of fresh resection material. A small piece was frozen for possible further analysis, and the rest of the material was formalin-fixed and paraffin-embedded for permanent sectioning. The tumor was composed of large cells that showed considerable variation in nuclear size and shape. The cell borders were indistinct, and the tumor cells exhibited anaplastic features with multiple nuclei and prominent nucleoli. The tumor had a prominent vascular network and geographic necrosis. Mitotic figures, up to 9 per 2 mm^2^, were easily identified **(Fig. 2)**.

**Figure 3:**
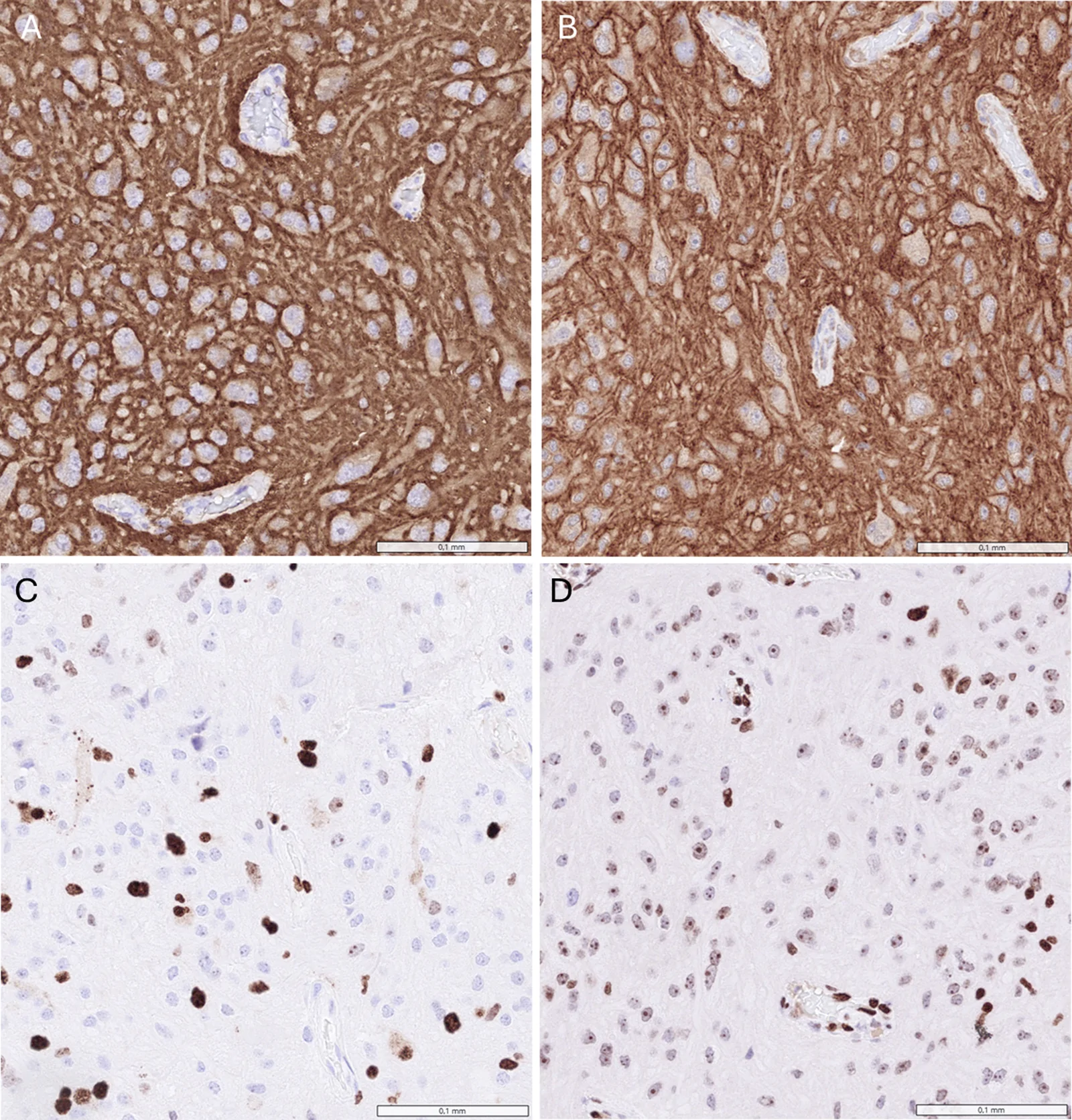
**Figure 3**. **A, B:** Synaptophysin (A) and SSTR2 (B) staining of tumor cells. **C:** Ki-67 staining indicated increased mitotic activity in tumor cells. **D:** H3K27me3 staining was abnormally negative in a subset of tumor cells.

Multiple immunohistochemical markers were ordered **(Fig. 3)**. The tumor cells were strongly positive for synaptophysin and somatostatin receptor 2 (SSTR2). The tumor cells also expressed chromogranin. Glial fibrillary acidic protein (GFAP) was negative in tumor cells but stained normal entrapped glial cells. Neuronal nuclear protein (NeuN) was repeatedly negative, whereas neuron-specific enolase (NSE) and CD56 were positive. S100 was positive in scattered tumor cells. Normal ATRX expression was retained in tumor cell nuclei, and p53 moderately stained scattered tumor cells. The proliferation index (Ki-67) was up to 20 % in hotspot areas. Interestingly, H3K27me3 staining was abnormal in a subset of tumor cells. BRAF V600E, EMA, IDH1 R132H, TTF1 (clone SP141), Olig2, INSM1, CD20, CD3, STAT6, and PRAME were negative in the tumor cells.

Representative areas were used for DNA extraction. Genomic profiling was performed using next-generation sequencing (NGS). The NGS panel is an in-house mutation panel designed for central nervous system (CNS) tumors and interrogates 32 genes commonly mutated in CNS tumors, including *ATRX*, *BCOR*, *BRAF*, *CTNNB1*, *EZH2*, *FGFR1*, *H3F3A*, *HIST1H3B*, *IDH1*, *IDH2*, and *TP53*. Furthermore, it detects amplifications in 24 genes, including *ALK*, *EGFR*, *FGFR1*, *MET*, *PDGFRA*, *PIK3CA*, *SMARCA4*, and *SMARCB1*. The panel recognizes point mutations and small deletions and insertions but does not detect gene fusions. In this case, the results of this panel were negative. Representative areas were also submitted for methylation profiling, but the tumor did not match to any entity in the Illumina MethylationEPIC v2.0 BeadChip/Epignostix CNS Tumor Methylation Classifier v12.8. Methylation profiling revealed an excess number of copies on chromosomes 7, 9, 11, 12, 13q, 17, and 21q.

After considering all available information, a diagnosis of atypical central neurocytoma was made. The patient’s case was discussed in a multidisciplinary neuro-oncology meeting, and a thorough follow-up with frequent MRI was recommended.

## Discussion

Central neurocytomas are rare intraventricular neuronal tumors that lack specific molecular alterations. The entity is currently defined as CNS WHO grade 2 by the WHO Classification of CNS Tumors, 5^th^ edition (WHO CNS5). Typically, central neurocytomas arise in the lateral ventricles of young adults, and the prognosis is generally favorable. In rare cases, however, central neurocytomas exhibit unusual histological features. These tumors have been labeled atypical central neurocytomas in the literature, but definitive and official criteria for atypical central neurocytomas are currently lacking.

The current WHO CNS5 states that anaplastic histological features, increased mitotic activity, microvascular proliferation, elevated Ki-67 (MIB-1) proliferation index, and necrosis can occur in rare cases, prompting designation of tumors with these features as atypical central neurocytomas. WHO CNS5 states that mitoses are exceptional and that the Ki-67 (MIB-1) proliferation index is usually low (< 2 %) in central neurocytomas. A high mitotic count and elevated Ki-67 (MIB-1) index have been shown to correlate with the prognosis of central neurocytomas (3). Other atypical histopathological features in central neurocytomas have not been reliably correlated with patient prognosis (3). As the Ki-67 (MIB-1) index seems to be the only reliable marker of aggressiveness, whether the presence of necrosis, anaplastic features, and microvascular proliferation should affect the grading of these tumors is still under debate. The WHO does not formally define the diagnostic criteria for “atypical” central neurocytoma, and although the term persists in the literature, it lacks standardized thresholds for diagnosis. Accordingly, the “atypical” designation remains non-standardized and is based on retrospective series rather than on consensus criteria.

Atypical cases reported previously have shown more aggressive behavior compared to conventional central neurocytomas. Byun et al. (2018) showed that a MIB-1 index of above 2 % was associated with increased risk of recurrence and more unfavorable 2-, 3-, and 5-year progression-free survival rates (4). Other studies have reported similar findings (10). Documentation of these features, however, generally suffers from interobserver and interlaboratory variability (3). Rare cases of cerebrospinal fluid (CSF) dissemination have also been reported (11). Although small reference cohorts complicate the evaluation of prognosis of atypical central neurocytomas, successful gross total resection is associated with significantly better outcomes than subtotal resection in small cohorts (3,12). Based on these reports, close radiologic follow-up is warranted in cases of atypical central neurocytomas.

Central neurocytomas are peculiar central nervous system tumors that lack defining molecular features. In an epigenetically defined group of central neurocytomas, hypomethylation at the *FGFR3* locus was reported in 97 % of cases (3). The only molecular finding in our case was multiple copy number alterations, which could suggest that atypical central neurocytoma could harbor genomic instability. Similar copy number alterations have been previously reported in central neurocytomas, although these findings have not shown any prognostic significance (3,8). Krech et al. (2025) reported that the most common copy number alternation in their cases was gain of chromosome 5 (3); similar to our case, they also reported gain of chromosome 17 in two cases and gain of chromosome 12 in one case (3). Still, most central neurocytomas do not seem to harbor any specific copy-number alternations, and central neurocytomas usually have balanced copy-number profiles (3).

The DNA methylation-based classification was not able to provide a diagnosis in our case, although the provided sample had a high tumor purity and was well preserved. Other technical aspects like poor probe quality could explain the failure of methylation-based classification, but the data received did not indicate any technical problems. The absence of a reliable methylation class assignment underscores the current limitations of molecular classification in rare intraventricular neuronal tumors. Our case further demonstrates that rare entities can prove notoriously difficult for the methylation classifier to assign a diagnosis.

The differential diagnosis of atypical central neurocytomas includes other intraventricular tumors such as ependymomas and other glial/glioneuronal tumors, choroid plexus papillomas, and rare cases of intraventricular meningiomas. Generally, tumors can be differentiated using immunohistochemistry. In difficult cases, documentation of molecular markers and methylation profiling are helpful, as many tumors harbor precise genetic alterations. In our case, anaplastic histological features were demonstrated, so the differential diagnosis of a high-grade CNS tumor is also justified, and adequate sampling is important for identification of possible glial components. High-grade gliomas with neuronal differentiation could show similar histology, but intraventricular location and total absence of convincing glial differentiation would be unexpected. The limitations of this case report include the exceeding rarity of atypical central neurocytomas and lack of long-term follow-up.

## Conclusion

In conclusion, we report a single case of a rare, atypical central neurocytoma with unusually aggressive histopathological features, including frank anaplasia and a high Ki-67 index. The tumor displayed multiple copy-number alterations. Methylation profiling was non-informative, highlighting its limitations in assigning a diagnosis to very uncommon tumors. The current WHO classification does not yet include diagnostic criteria for atypical central neurocytomas, even though rare reports of these tumors exist. Our case also highlights the importance of an integrated clinicopathological evaluation.

## Conflicts of interest statement

The authors declare no conflicts of interest.

## AI contribution

We declare that we have utilized Paperpal (https://paperpal.com/) solely as a writing and language enhancement tool in the preparation of this article. Its use was strictly limited to improving the grammar, sentence structure, clarity, and coherence of the written text.

## References

[R1] Stapleton CJ, Walcott BP, Kahle KT, Codd PJ, Nahed BV, Chen L, Robison NJ, Delalle I, Goumnerova LC, Jackson EM. Diffuse central neurocytoma with craniospinal dissemination. J Clin Neurosci. 2012 Jan;19(1):163–6. Epub 2011 Nov 15. 10.1016/j.jocn.2011.07.016 22088950

[R2] Ando K, Ishikura R, Morikawa T, Nakao N, Ikeda J, Matsumoto T, Arita N. Central neurocytoma with craniospinal dissemination. Magn Reson Med Sci. 2002 Nov 1;1(3):179–82. 10.2463/mrms.1.179 16082142

[R3] Krech M, Muench A, Teichmann D, Kuzman P, Suwala AK, Ippen FM, Müther M, Weber KJ, Wenger-Alakmeh K, Onken J, Vajkoczy P, Behling F, May SA, Ntoulias G, Krauss JK, Atallah O, Esmaeilzadeh M, Mueller WC, Heppner FL, Radbruch H, Dittmayer C, Stenzel W, Koch A, Capper D, Kaul D, Paulus W, Plate KH, Steinbach JP, Czabanka M, Beschorner R, von Deimling A, Bockmayr M, Neumann JE, Brandner S, Krieger T, Hartmann C, Thomas C, Schweizer L. Outcome-associated factors in a molecularly defined cohort of central neurocytoma. Acta Neuropathol. 2025 Jun 11;149(1):61. 10.1007/s00401-025-02894-3 PMC12158839 40498174

[R4] Byun J, Hong SH, Yoon MJ, Kwon SM, Cho YH, Kim JH, Kim CJ. Prognosis and treatment outcomes of central neurocytomas: clinical interrogation based on a single center experience. J Neurooncol. 2018 Dec;140(3):669–677. Epub 2018 Sep 17. 10.1007/s11060-018-2997-z 30225773

[R5] Leenstra JL, Rodriguez FJ, Frechette CM, Giannini C, Stafford SL, Pollock BE, Schild SE, Scheithauer BW, Jenkins RB, Buckner JC, Brown PD. Central neurocytoma: management recommendations based on a 35-year experience. Int J Radiat Oncol Biol Phys. 2007 Mar 15;67(4):1145–54. Epub 2006 Dec 21. 10.1016/j.ijrobp.2006.10.018 17187939

[R6] Capper D, Stichel D, Sahm F, Jones DTW, Schrimpf D, Sill M, Schmid S, Hovestadt V, Reuss DE, Koelsche C, Reinhardt A, Wefers AK, Huang K, Sievers P, Ebrahimi A, Schöler A, Teichmann D, Koch A, Hänggi D, Unterberg A, Platten M, Wick W, Witt O, Milde T, Korshunov A, Pfister SM, von Deimling A. Practical implementation of DNA methylation and copy-number-based CNS tumor diagnostics: the Heidelberg experience. Acta Neuropathol. 2018 Aug;136(2):181–210. Epub 2018 Jul 2. 10.1007/s00401-018-1879-y PMC6060790 29967940

[R7] Kim H, Lee K, Shim YM, Kim EE, Kim SK, Phi JH, Park CK, Choi SH, Park SH. Epigenetic Alteration of H3K27me3 as a Possible Oncogenic Mechanism of Central Neurocytoma. Lab Invest. 2023 Aug;103(8):100159. Epub 2023 Apr 22. 10.1016/j.labinv.2023.100159 37088465

[R8] Korshunov A, Sycheva R, Golanov A. Recurrent cytogenetic aberrations in central neurocytomas and their biological relevance. Acta Neuropathol. 2007 Mar;113(3):303–12. Epub 2006 Nov 23. 10.1007/s00401-006-0168-3 17123091

[R9] Kalawi AZ, Malicki DM, Abdullaev Z, Pratt DW, Quezado M, Aldape K, Elster JD, Paul MR, Khanna PC, Levy ML, Crawford JR. The role of methylation profiling in histologically diagnosed neurocytoma: a case series. J Neurooncol. 2022 Sep;159(3):725–733. Epub 2022 Aug 22. 10.1007/s11060-022-04117-1 PMC9477906 35994156

[R10] Imber BS, Braunstein SE, Wu FY, Nabavizadeh N, Boehling N, Weinberg VK, Tihan T, Barnes M, Mueller S, Butowski NA, Clarke JL, Chang SM, McDermott MM, Prados MD, Berger MS, Haas-Kogan DA. Clinical outcome and prognostic factors for central neurocytoma: twenty year institutional experience. J Neurooncol. 2016 Jan;126(1):193–200. 10.1007/s11060-015-1959-y PMC4826306 26493740

[R11] Juratli TA, Geiger K, Leimert M, Schackert G, Kirsch M. Atypical Central Neurocytoma with Recurrent Spinal Dissemination over a Period of 20 Years: A Case Report and Review of the Literature. Case Rep Neurol Med. 2013;2013:925647. Epub 2013 Jun 9. 10.1155/2013/925647 PMC3690632 23840986

[R12] AbdelBari Mattar M, Shebl AM, Toson EA. Atypical Central Neurocytoma: An Investigation of Prognostic Factors. World Neurosurg. 2021 Feb;146:e184–e193. Epub 2020 Oct 19. 10.1016/j.wneu.2020.10.068 33091649

